# Special Issue “Sugar Transport, Metabolism and Signaling in Plants”

**DOI:** 10.3390/ijms24065655

**Published:** 2023-03-16

**Authors:** Soulaiman Sakr

**Affiliations:** Institut Agro, University of Angers, INRAE, IRHS, SFR QUASAV, 49000 Angers, France; soulaiman.sakr@agrocampus-ouest.fr

Sucrose and its derivative hexoses are key metabolites of the plant metabolism, structural units of cell walls and stored reserves (e.g., starch), and also crucial metabolic signals that interplay with other metabolic, hormonal and environmental cues to fine-tune plant growth and development [1,2,3]. Their partitioning over short or long distances is crucial for the establishment of source–sink organ relationships and agronomical plant performance. Although stem nodes are considered to act as hubs in the control of nutrient partitioning from photosynthetic leaves to non-photosynthetic vegetative and reproductive organs, many important details remain obscure, arousing considerable interest among researchers. To bring new insights into this issue, ref. [4] investigated the physiological characteristics of maize stem nodes associated with whole-plant carbon partitioning in response to two regimes of nitrogen (N) availability. They first demonstrated that low nitrogen availability led to delayed plant growth and decreased sugar levels in the leaves compared with N-sufficient plants, while stem nodes displayed a stably higher sugar content, suggesting a facilitating role for nodes in whole-plant carbon partitioning. Additional experiments on stem nodes revealed that low N availability led to elevated contents of soluble sugars along with the high activity of several sugar transporters. This accumulation was developmental-stage-dependent: sugar levels were higher in the bottom part of the nodes than in their upper part. Overall, these results highlight the role of stem nodes in facilitating long-distance sugar transport in maize. It will be interesting to understand the mechanisms involved in this process in the near future. Within this context, ref. [5] reported that the remobilization of non-structural carbohydrates (NSCs) in rice stems during grain filling might be related to the stem-located trehalose 6P (T6P) complex and *SnRK1* (sucrose non-fermenting-related kinase protein), two main components of the sugar-signaling pathways [1]. This study was carried out on two large-panicle rice varieties (CJ03 and W1844) differing in the sink strength of their inferior spikelet (IS). The IS has a poor grain filling capacity and thereby represents a suitable model for examining the relationship between sink strength and sugar availability in the stem. These authors showed that the IS grains of the W1844 cultivar displayed a stronger sink strength than those of CJ03. Moreover, W1844 outperformed CJ03 in terms of the efficiency of carbon reserve remobilization in the stem. By monitoring the dynamics of the T6P content, the expression of trehalose-metabolizing enzymes and the expression of different sub-units of *SnRK1*, these authors proposed that NSC remobilization in the stem may follow a “feast-or-famine”-like model, which fits perfectly well with the fluctuating source–sink relationship during the grain filling stage. In addition, this remobilization could be mainly driven by the sink strength of the panicle. These two works lay a solid foundation for further exploring the relationship between the stem sugar signaling network, the stem carbon metabolism and sink strength, and open up promising research prospects on the molecular regulatory network and the way sink organs remotely control carbon remobilization in the stem.

Sugar transport across the plant involves a variety of transporters gathered into three main families: monosaccharide transporter-like (MST) genes, sucrose transporters (SUTs/SUCs) and sugar-will-eventually-be-exported transporters (SWEETS) [6]. In addition to its well-known role as a carbon source and building block, sugar serves as an osmotic agent to confer plants resistance to abiotic stress. Two research papers [7,8] elegantly demonstrated the crucial role of the SWEET gene family in the cold tolerance of plants. In Longan (*Dimocarpus longan*), a subtropic fruit tree with high economic value and sensitivity to cold, ref. [7] carried out a huge study consisting of identifying 20 longan SWEET (referred to as DiSWEET) genes and characterizing their phylogenetic relationships, structures, *cis*-acting elements and tissue-expression patterns. They found that this transporter family is divided into four clades with conserved motifs and similar exon–intron organization. More notably, and based on the genetic approach, they found evidence that only *DiSWEET1* was greatly induced by cold, supporting its contribution to the adaptive response of longan to low temperature. Similarly, ref. [8] highlighted the role of *CsSWEET2* in the cold tolerance of cucumber (*Cucumis sativus*). *CsSWEET2* encodes an energy-independent hexose/H^+^ uniporter and is localized on the plasma membrane and endoplasmic reticulum. Interestingly, its overexpression in Arabidopsis plants resulted in the accumulation of soluble sugars (glucose and fructose) and resistance to cold stress. All these results point out the functional diversity of SWEET sugar transporters in plant physiology and open new avenues of research on the molecular network orchestrating SWEET upregulation by environmental factors.

Sugar-signaling pathways interact with hormonal pathways in several biological contexts and throughout the life cycle of the plant [1,2,9,10,11]. Since the discovery of the Abscisic acid, Stress, Ripening (ASR) protein in plants, several physiological functions have been attributed to it during the plant life cycle and in response to adverse conditions [12,13,14]. Previous studies indicated that grape ASR (*Vitis vinifira* maturation, stress, abscisic-acid induced; *VvMSA*) transcriptionally regulates the hexose transporter gene *Vitis vinifera* Hexose Transporter 1 (*VvHT1*), and its expression is at the crosstalk of glucose and ABA signaling [15]. To gain an initial understanding of the position of *VvMSA* in glucose signaling, ref. [16] used a comprehensive approach including pharmacological, biochemical, molecular and genetic strategies in *VvMSA* gain- and loss-of-function embryogenic and non-embryogenic grapevine cells. The choice of these two models is crucial for discriminating between glucose-sensitive signaling and glucose-metabolism-dependent processes. These authors clearly highlighted that *VvMSA* acts as a transcriptional regulator of the glucose transporter gene *VvHT1* in glycolysis-dependent glucose signaling and regulates the interplay between glucose metabolism, transport and signaling. These results shed new light on the regulatory network of the ASR family and fill a gap in our understanding of the regulation of this complex protein family.

Among the sugar-related signaling pathways, the oxidative pentose phosphate pathway (OPPP) remains the least investigated, even though it is involved in sugar-mediated nitrate and sulfate transporters in *Rosa* sp. roots [17] and bud outgrowth [18,19]. In their review, ref. [20] focused on glucose-6-phosphate dehydrogenase (G6PDH), which catalyzes the rate-limiting step, driving carbon flow through the OPPP, which might play a direct and/or indirect role in the sugar-signaling pathway. G6PDH catalyzes a metabolic hub between glycolysis and the pentose phosphate pathway (PPP). The review draws a concise and comprehensive picture of the diversity of plant G6PDHs and their role in seed germination, nitrogen assimilation, plant branching and plant response to abiotic stress. It constitutes a solid basis for defining future research directions to improve our knowledge of G6PDHs in plant physiology and to integrate this hidden player in plant performance. 

Concluding remarks. This Special Issue “Sugar Transport, Metabolism and Signaling in Plants” is an excellent opportunity to gather a variety of scientific research works involving in-depth investigations. Each of them sheds new and interesting light on sugar-related topics, including the sink–source relationship, focusing on the role of the stem node, the role of SWEET in plant stress responses and the hitherto neglected sugar-signaling pathway within the OPPP. They open up promising avenues for uncovering the molecular regulatory network behind this physiological function and integrating this knowledge within the overall functioning of the plant.

## References

[1] Sakr S., Wang M., Dédaldéchamp F., Perez-Garcia M.-D., Ogé L., Hamama L., Atanassova R. (2018). The Sugar-Signaling Hub: Overview of Regulators and Interaction with the Hormonal and Metabolic Network. Int. J. Mol. Sci..

[2] Wang M., Le Gourrierec J., Jiao F., Demotes-Mainard S., Perez-Garcia M.-D., Ogé L., Hamama L., Crespel L., Bertheloot J., Chen J. (2021). Convergence and Divergence of Sugar and Cytokinin Signaling in Plant Development. Int. J. Mol. Sci..

[3] Jeandet P., Formela-Luboińska M., Labudda M., Morkunas I. (2022). The Role of Sugars in Plant Responses to Stress and Their Regulatory Function during Development. Int. J. Mol. Sci..

[4] Zhao Y., Ning P., Feng X., Ren H., Cui M., Yang L. (2022). Characterization of Stem Nodes Associated with Carbon Partitioning in Maize in Response to Nitrogen Availability. Int. J. Mol. Sci..

[5] Jiang Z., Chen Q., Chen L., Liu D., Yang H., Xu C., Hong J., Li J., Ding Y., Sakr S. (2022). Sink Strength Promoting Remobilization of Non-Structural Carbohydrates by Activating Sugar Signaling in Rice Stem during Grain Filling. Int. J. Mol. Sci..

[6] Doidy J., Vidal U., Lemoine R. (2019). Sugar Transporters in Fabaceae, Featuring SUT MST and SWEET Families of the Model Plant *Medicago truncatula* and the Agricultural Crop *Pisum sativum*. PLoS ONE.

[7] Fang T., Rao Y., Wang M., Li Y., Liu Y., Xiong P., Zeng L. (2022). Characterization of the SWEET Gene Family in Longan (Dimocarpus longan) and the Role of DlSWEET1 in Cold Tolerance. Int. J. Mol. Sci..

[8] Hu L., Zhang F., Song S., Yu X., Ren Y., Zhao X., Liu H., Liu G., Wang Y., He H. (2022). CsSWEET2, a Hexose Transporter from Cucumber (*Cucumis sativus* L.), Affects Sugar Metabolism and Improves Cold Tolerance in Arabidopsis. Int. J. Mol. Sci..

[9] Rabot A., Portemer V., Péron T., Mortreau E., Leduc N., Hamama L., Coutos-Thévenot P., Atanassova R., Sakr S., Le Gourrierec J. (2014). Interplay of Sugar, Light and Gibberellins in Expression of Rosa hybrida Vacuolar Invertase 1 Regulation. Plant Cell Physiol..

[10] Jurczyk B., Pociecha E., Janowiak F., Dziurka M., Kościk I., Rapacz M. (2021). Changes in Ethylene, ABA and Sugars Regulate Freezing Tolerance under Low-Temperature Waterlogging in *Lolium perenne*. Int. J. Mol. Sci..

[11] Wang M., Ogé L., Pérez Garcia M.D., Launay-Avon A., Clément G., Le Gourrierec J., Hamama L., Sakr S. (2022). Antagonistic Effect of Sucrose Availability and Auxin on Rosa Axillary Bud Metabolism and Signaling, Based on the Transcriptomics and Metabolomics Analysis. Front. Plant Sci..

[12] Golan I., Dominguez P.G., Konrad Z., Shkolnik-Inbar D., Carrari F., Bar-Zvi D. (2014). Tomato ABSCISIC ACID STRESS RIPENING (ASR) Gene Family Revisited. PLoS ONE.

[13] Zhang J., Zhu Q., Yu H., Li L., Zhang G., Chen X., Jiang M., Tan M. (2019). Comprehensive Analysis of the Cadmium Tolerance of Abscisic Acid-, Stress- and Ripening-Induced Proteins (ASRs) in Maize. Int. J. Mol. Sci..

[14] Lin R., Zou T., Mei Q., Wang Z., Zhang M., Jian S. (2021). Genome-Wide Analysis of the Late Embryogenesis Abundant (LEA) and Abscisic Acid-, Stress-, and Ripening-Induced (ASR) Gene Superfamily from *Canavalia rosea* and Their Roles in Salinity/Alkaline and Drought Tolerance. Int. J. Mol. Sci..

[15] Çakir B., Agasse A., Gaillard C., Saumonneau A., Delrot S., Atanassova R. (2003). A Grape ASR Protein Involved in Sugar and Abscisic Acid Signaling. Plant Cell.

[16] Parrilla J., Medici A., Gaillard G., Verbeke J., Gibon Y., Rolin D., Laloi M., Finkelstein R.R., Atanassova R. (2022). Grape ASR Regulates Glucose Transport, Metabolism and Signaling. Int. J. Mol. Sci..

[17] Lejay L., Wirth J., Pervent M., Cross J.M.F., Tillard P., Gojon A. (2008). Oxidative Pentose Phosphate Pathway-Dependent Sugar Sensing as a Mechanism for Regulation of Root Ion Transporters by Photosynthesis. Plant Physiol..

[18] Wang M., Ogé L., Voisine L., Perez-Garcia M.D., Jeauffre J., Hibrand Saint-Oyant L., Grappin P., Hamama L., Sakr S. (2019). Posttranscriptional Regulation of *RhBRC1* (Rosa hybrida BRANCHED1) in Response to Sugars is Mediated via its Own 3′ Untranslated Region, with a Potential Role of RhPUF4 (Pumilio RNA-Binding Protein Family). Int. J. Mol. Sci..

[19] Wang M., Pérez-Garcia M.D., Davière J.M., Barbier F., Ogé L., Gentilhomme J., Voisine L., Péron T., Launay-Avon A., Clément G. (2021). Outgrowth of the axillary bud in rose is controlled by sugar metabolism and signalling. J. Exp. Bot..

[20] Jiang Z., Wang W., Nicolas M., Ogé L., Pérez-Garcia M.D., Crespel L., Li G., Ding Y., Le Gourrierec J., Grappin P. (2022). Glucose-6-Phosphate Dehydrogenases: The Hidden Players of Plant Physiology. Int. J. Mol. Sci..

